# Exendin-4 and Liraglutide Attenuate Glucose Toxicity-Induced Cardiac Injury through mTOR/ULK1-Dependent Autophagy

**DOI:** 10.1155/2018/5396806

**Published:** 2018-04-19

**Authors:** Wei Yu, Wenliang Zha, Jun Ren

**Affiliations:** ^1^Department of Pharmacology, School of Pharmacy, Hubei University of Science and Technology, Xianning, Hubei 437100, China; ^2^Center for Cardiovascular Research and Alternative Medicine, University of Wyoming College of Health Sciences, Laramie, WY 82071, USA; ^3^Department of Surgery, Clinic Medical College, Hubei University of Science and Technology, Xianning, Hubei 437100, China; ^4^Department of Cardiology, Fudan University Zhongshan Hospital, Shanghai 210032, China

## Abstract

Mitochondrial injury and defective autophagy are common in diabetic cardiomyopathy. Recent evidence supports benefits of glucagon-like peptide-1 (GLP-1) agonists exendin-4 (Exe) and liraglutide (LIRA) against diabetic cardiomyopathy. This study was designed to examine the effect of Exe and LIRA on glucose-induced cardiomyocyte and mitochondrial injury, oxidative stress, apoptosis, and autophagy change. Cardiomyocytes isolated from adult mice and H9c2 myoblast cells were exposed to high glucose (HG, 33 mM) with or without Exe or LIRA. Cardiac contractile properties were assessed including peak shortening, maximal velocity of shortening/relengthening (±d*L*/d*t*), time to PS, and time-to-90% relengthening (TR_90_). Superoxide levels, apoptotic proteins such as cleaved caspase-3, Bax, and Bcl-2, and autophagy proteins including Atg5, p62, Beclin-1, LC3B, and mTOR/ULK1 were evaluated using Western blot. Mitochondrial membrane potential (MMP) changes were assessed using JC-1, and autophagosomes were determined using GFP-LC3. Cardiomyocyte exposure to HG exhibited prolonged TR_90_ associated with significantly decreased PS and ±d*L*/d*t*, the effects of which were partly restored by GLP-1 agonists, the effects of which were negated by the mTOR activator 3BDO. H9c2 cell exposure to HG showed increased intracellular ROS, apoptosis, MMP loss, dampened autophagy, and elevated p-mTOR and p-ULK1, the effects of which were nullified by the GLP-1 agonists. These results suggested that GLP-1 agonists rescued glucose toxicity likely through induction of mTOR-dependent autophagy.

## 1. Introduction

Diabetes mellitus is becoming a major health threat as the International Diabetes Federation (IDF) predicts a startling number of 642 million patients with diabetes by the year of 2040 [1]. This chronic metabolic disease can steadily trigger a cascade of long-term severe complications such as cardiovascular diseases, peripheral vascular diseases, and central nervous system and kidney diseases [2–5]. Among these comorbidities, diabetic cardiomyopathy occurs independent of macro- and micro-coronary artery diseases and other cardiovascular diseases and imposes a high risk for cardiovascular morbidity and mortality [6]. The major pathological manifestations of diabetic cardiomyopathy include hypertrophy, ventricular dilatation, and compromised contractile function, which may be attributed to apoptosis and interstitial fibrosis, leading to ventricular remodeling [7, 8]. Previous studies from our lab and others have depicted a number of pathophysiological factors for the onset and development of diabetic cardiomyopathy including glucose and lipid toxicity, inflammation, oxidative stress, mitochondrial injury, interstitial fibrosis, apoptosis, and dysregulated autophagy [9–12]. Nevertheless, the precise molecular mechanism behind diabetic cardiomyopathy remains obscure.

Autophagy denotes a cellular degradation process for long-lived or damaged proteins and cytoplasmic organelles, through which these cytoplasmic proteins are degraded and recycled by lysosomes [13]. Autophagy plays a pivotal role for cardiac homeostasis in both physiological and pathological conditions [14]. Constitutive autophagy helps to sustain a balance between the synthesis, degradation, and subsequent recycling of cellular elements. Recent studies have indicated that levels of autophagy may be suppressed in diabetes, leading to the development of diabetic cardiomyopathy [15]. To this end, there is a growing interest in the administration of an autophagy inducer as a therapeutic approach in diabetes although many of the autophagy inducers suffer from pitfalls such as toxicity and undesired off-target effects.

Glucagon-like peptide-1 (GLP-1), synthesized and secreted from L-cells of the small intestine, is a 30-amino acid peptide with potent biological effects. The GLP-1 receptor is a G protein-coupled receptor broadly expressed in peripheral tissues including islet cells, kidney, lung, brain, and gastrointestinal tract [16]. Many peripheral tissues including the heart possess a GLP-1 receptor reminiscent of those found in pancreatic cells [16]. Clinical and experimental evidence has shown the utility of GLP-1 at the time of reperfusion in reducing myocardial infarct size, mitigating ischemic-reperfusion injury, and improving cardiac functions [17, 18]. Moreover, recent date suggested that GLP-1, its analogues, and receptor agonists are capable of benefiting diabetes, diabetic retinopathy, nephropathy, and peripheral neuropathy [19–21]. More interestingly, the GLP-1 receptor agonist exendin-4 (Exe) offers protective effects against type 2 diabetes-induced brain injury via autophagy induction [22]. However, whether GLP-1 receptor agonists play any role in diabetic cardiomyopathy remains unknown. Therefore, this study was designed to evaluate the effect of GLP-1 agonists Exe and liraglutide (LIRA) on high glucose-induced cardiomyocyte contractile dysfunction, mitochondrial injury, oxidative stress, apoptosis, and changes in autophagy.

## 2. Materials and Methods

### 2.1. Cardiomyocyte Isolation and Mechanics

All animal procedures used here were approved by the Animal Care and Use Committee at the University of Wyoming (Laramie, WY). In brief, hearts were removed rapidly from adult wild-type mice sedated with ketamine (80 mg/kg, ip) and xylazine (12 mg/kg, ip) and perfused with Krebs-Henseleit bicarbonate (KHB) solution consisting of (in mM) 118 NaCl, 4.7 KCl, 1.2 MgSO_4_, 1.2 KH_2_PO_4_, 25 NaHCO_3_, 10 HEPES, and 11.1 glucose. Hearts were digested with Liberase Blendzyme™ (Roche Diagnostics, Indianapolis, IN) for 15 min. After removal and mincing of the left ventricle, Ca^2+^ was added back to a final concentration of 1.25 mM. Cardiomyocytes with no spontaneous contractions and clear edges were used for shortening. The IonOptix SoftEdge system (IonOptix, Milton, MA) was employed to assess the mechanical properties of isolated myocytes. Myocytes were mounted on the stage of an Olympus IX70 microscope in contractile buffer containing (in mM) 131 NaCl, 4 KCl, 1 CaCl_2_, 1 MgCl_2_, 10 glucose, and 10 HEPES. Myocytes were stimulated at 0.5 Hz with cell shortening and relengthening evaluated using the following indices: peak shortening (PS), time to peak shortening (TPS), time to 90% relengthening (TR_90_), and maximal velocities of shortening/relengthening (±d*L*/d*t*) [11]. To evaluate the effect of the GLP-1 agonist on glucose toxicity-induced changes in cardiac function, cells were cultured for 4 hours in a KHB solution containing 33 mM or 5.5 mM glucose (designated as high glucose and normal glucose) with or without Exe (10 nM) or LIRA (100 nM) prior to the assessment of cardiomyocyte mechanical properties.

### 2.2. Cell Culture and Treatment

Rat cardiomyocyte-derived cell line H9c2 was purchased from the American Type Culture Collection (ATCC, CRL-1446™) and was cultured in DMEM supplemented with 10% FBS, 1% penicillin, and streptomycin at 37°C in a humidified atmosphere (5% CO_2_ and 95% air). When cells reached confluence at 70%–80%, they were exposed to normal glucose (NG, 5.5 mM) or high glucose (HG, 33 mM) for 48 hours in the absence or presence of Exe (10 nM) or LIRA (100 nM). To assess whether high glucose affects autophagy, H9c2 cells were exposed with high glucose medium in the absence or presence of Exe (10 nM) or LIRA (100 nM) in the presence or absence of the autophagy inducer rapamycin (100 nM) or the autophagy inhibitor 3-methyladenine (3-MA, 10 mM) [23]. To assess the role of mTOR in Exe- and LIRA-induced response, if any, against high glucose-induced cardiomyocyte mechanical dysfunction, murine cardiomyocytes were exposed with high glucose medium in the absence or presence of Exe (10 nM) or LIRA (100 nM) for 4 hours with or without the mTOR activator [3-benzyl-5-((2-nitrophenoxy) methyl)-dihydrofuran-2(3H)-one (3BDO),120 *μ*M] [24] prior to assessment of mechanical function.

### 2.3. Intracellular Reactive Oxygen Species (ROS) Measurement

Intracellular superoxide anions were measured using the dihydroethidium (DHE) fluorescence probe. The cells were incubated in a light-impermeable chamber at 37°C for 30 min after application of 10 *μ*M DHE (Life Technologies, USA) and then were cultured with 5 *μ*g/ml DAPI (Sigma, USA) for 5 min. The images of H9c2 cardiomyocytes were captured and analyzed immediately under a fluorescence microscope (Olympus BX51, Japan). Production of reactive oxygen species (ROS) in cultured cells was assessed by 5-(6)-chloromethyl-2′,7′-dichlorodihydrofluorescein diacetate (CM-H2DCFDA) molecular probe staining. In brief, H9c2 cells were loaded with 1 *μ*M H_2_DCFDA at 37°C for 30 min. The cells were rinsed with PBS, and the fluorescence intensity was then detected by a fluorescent microplate reader (Molecular Devices, Sunnyvale, CA) at an excitation wavelength of 480 nm and an emission wavelength of 530 nm [25].

### 2.4. Measurement of MitoSOX

The mito-ROS level was assessed using the MitoSOX™ Red mitochondrial superoxide indicator (Life Technologies, USA) according to the manufacturer's instructions. Briefly, after treatment, the cells were washed three times with PBS and incubated with 5 *μ*M MitoSOX for 30 min in the dark. The level of mito-ROS was detected by a fluorescent microplate reader (Molecular Devices, Sunnyvale, CA) at 485 nm for excitation and 590 nm for emission [26].

### 2.5. Measurement of Mitochondrial Membrane Potential (MMP, ΔΨm)

Changes of MMP was measured using the fluorescent dye, 5′,6,6′-tetrachloro-1,1′,3,3′-tetraethyl-imidacarbocyanine iodide (JC-1 fluorochrome, Sigma, USA) as described [27]. Briefly, after the treatment of the cells, 5 *μ*M JC-1 staining was added to the cells and incubated in the dark for 30 min at 37°C. PBS buffer was used to wash H9c2 cells for three times and subsequently examined under a fluorescent microplate reader (Spectra Max Gemini XS) at 530 nm (monomer form of JC-1, green) and at 590 nm (aggregate form of JC-1, red). The mitochondrial uncoupler carbonyl cyanide m-chlorophenyl hydrazone (CCCP, 10 *μ*M) was used as the positive control [28]. In addition, cells labeled with JC-1 were observed using a fluorescence microscope (Olympus BX51, Japan).

### 2.6. LC3B-GFP-Adenovirus Infection in H9c2

H9c2 cells were assessed by autophagy using GFP fluorescence [29]. An adenovirus containing a GFP-LC3 construct was provided by Dr. Cindy Miranti from the University of Arizona (Phoenix, AZ) and was propagated using the HEK293 cell line. Cells were transfected with GFP-LC3 adenovirus for 8 hours and then refreshed with normal medium. After 48 hours, cells were visualized for autophagy using fluorescence microscopy and were treated with either normal or high glucose in the absence or presence of GLP-1 agonists or the autophagy inhibitor 3-MA. Rapamycin was used as the positive control. For autophagy evaluation, cells were captured under a fluorescence microscope (Olympus BX51) and the percentage of GFP-LC3-positive cells showing numerous GFP-LC3 puncta (>10 dots/cell) was achieved as described previously [29]. Approximately 300–400 cells in each group were calculated in at least three independent experiments.

### 2.7. Western Blot Analysis

Cardiomyocytes were homogenized in a lysis buffer containing RIPA (Cell Signaling Technology, Danvers, MA), 1% NaF, 1% Na_3_VO_4_, and 1% protease inhibitor cocktail. Supernatants were separated after centrifugation at 12,000*g* for 15 min at 4°C. The protein levels of supernatant were quantified using the BSA protein assay. Equal amounts (30 mg protein/lane) of proteins were separated on 10% or 12% SDS-polyacrylamide gels and transferred to nitrocellulose membranes. Membranes were blocked and incubated overnight at 4°C with the following antibodies: p-mTOR (Ser^2448^), mTOR, p-ULK1 (Ser^757^), ULK1, Bcl-2, Bax, cleaved caspase-3, Atg5, P62, Beclin-1, LC3B, and GAPDH (Cell Signaling). Membranes were incubated for 1 hour at 37°C with a horseradish peroxidase-conjugated secondary antibody. Blots were assessed by the luminescence method. The Quantity One software (Bio-Rad, version 4.4.0, ChemiDoc XRS) was used for analysis quantification of immunoblots [29].

### 2.8. Statistical Analysis

Data were mean ± SEM. All statistical analyses were subjected to one-way ANOVA, and a *p* value less than 0.05 was considered to be significant.

## 3. Results

### 3.1. Effect of Exendin-4 and Liraglutide on Cardiomyocyte Shortening

**Figure 1:**
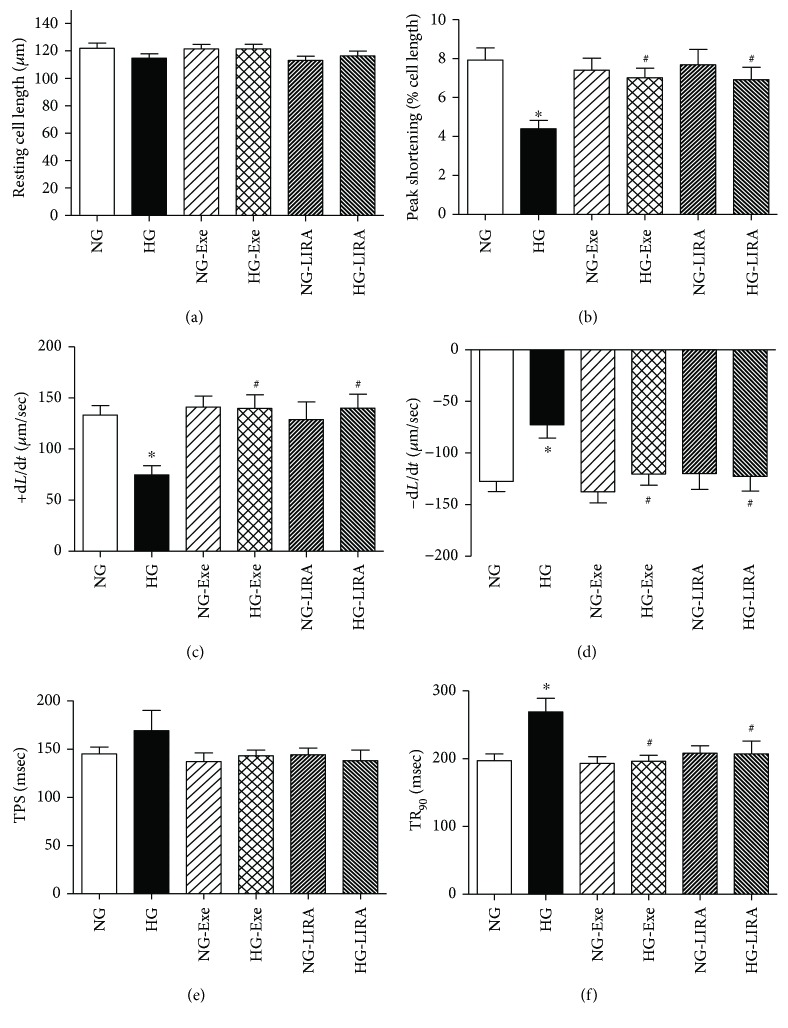
Effect of exendin-4 (Exe) and liraglutide (LIRA) on cardiomyocyte shortening in ventricular myocytes isolated from adult mouse hearts: (a) resting cell length; (b) peak shortening (PS); (c) maximal velocity of shortening (+d*L*/d*t*); (d) maximal velocity of relengthening (−d*L*/d*t*); (e) time to PS (TPS); (f) time-to-90% relengthening (TR_90_). Mean ± SEM, *n* = 63–66 cells per group, ^∗^*p* < 0.05 versus the NG group; ^#^*p* < 0.05 versus the HG group.

Short-term exposure (4 hours) of high glucose (33 mM) did not affect the resting cell length in murine cardiomyocytes. However, high glucose incubation suppressed peak cell shortening (PS), maximal velocity of shortening/relengthening (+d*L*/d*t* and −d*L*/d*t*), and prolonged time-to-90% relengthening (TR_90_) without affecting time-to-peak shortening (TPS). Although Exe and LIRA themselves did not affect these cardiomyocyte mechanical functions, they effectively rescued against glucose toxicity-induced changes in PS, ±d*L*/d*t*, and TR90 without affecting TPS and resting cell length (Figure 1).

### 3.2. Exendin-4 and Liraglutide Attenuated High Glucose-Induced ROS/O_2_^−^ Production and Downregulation of Nrf2

**Figure 2:**
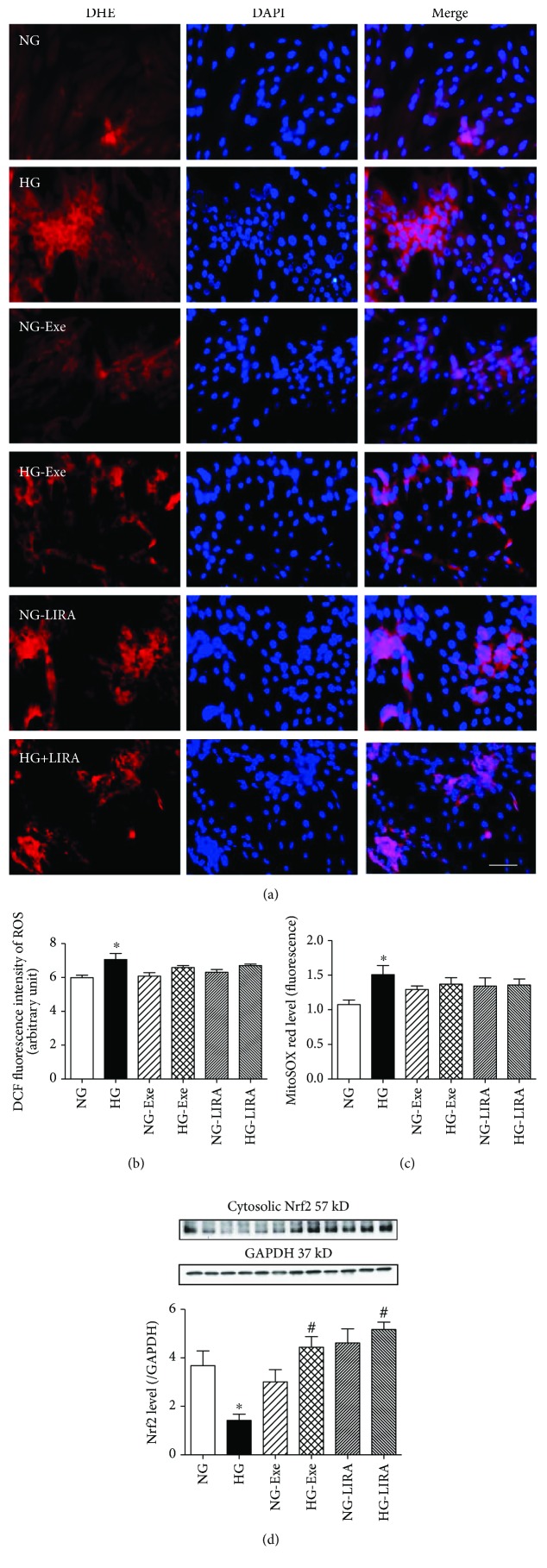
Effect of exendin-4 (Exe) and liraglutide (LIRA) on accumulation of ROS and mitochondrial O_2_^−^ as well as antioxidant Nrf2: (a) representative images of DCF staining depicting the effect of Exe and LIRA on high glucose-induced ROS production in H9c2 cells, scale bar = 50 *μ*m; (b) pooled data of DCF quantification, *n* = 7; (c) quantification of MitoSOX red intensity, *n* = 7; (d) levels of Nrf2 normalized to GAPDH. Inset: representative gel blots of Nrf2 and GAPDH using specific antibodies, *n* = 4, independent cell cultures per group, mean ± SEM, ^∗^*p* < 0.05 versus the NG group and ^#^*p* < 0.05 versus the HG group.

ROS plays an essential role in glucose toxicity and diabetic cardiomyopathy [30, 31]. Here we examined levels of ROS and O_2_^−^ using fluorescence techniques. As shown in Figure 2(a), O_2_^−^ production assessed using DHE fluorescence was significantly elevated in the high glucose group compared with the normal glucose group. Although GLP-1 agonists did not affect O_2_^−^ levels in normal glucose groups, they attenuated high glucose-induced elevation in O_2_^−^ levels. To further verify these results, H9c2 cells were stained with the specific oxidation-sensitive fluorescent dye DCF; high glucose significantly enhanced the relative fluorescent intensity of DCF, corresponding to an increased ROS level, the effects of which were attenuated by either Exe or LIRA. Neither GLP-1 agonist had any effect on DCF fluorescence intensity themselves (Figure 2(b)).

Earlier evidence suggested a pivotal role for mitochondrial ROS in the development of diabetic cardiomyopathy [31]. As demonstrated in Figure 2(c), mitochondrial O_2_^−^ evaluated using MitoSOX Red revealed that high glucose facilitated the generation of mitochondrial O_2_^−^, the effect of which was partly reversed by Exe and LIRA. Neither GLP-1 agonist exerted any notable effect on mitochondrial O_2_^−^ themselves. We went on to examine the changes of the cytosolic antioxidant Nrf2 in H9c2 cells. As shown in Figure 2(d), high glucose overtly downregulated the Nrf2 expression, the effects of which were alleviated by either Exe or LIRA, with little effect from the GLP-1 agonists themselves.

### 3.3. Exendin-4 and Liraglutide Inhibited High Glucose-Induced Apoptosis

**Figure 3:**
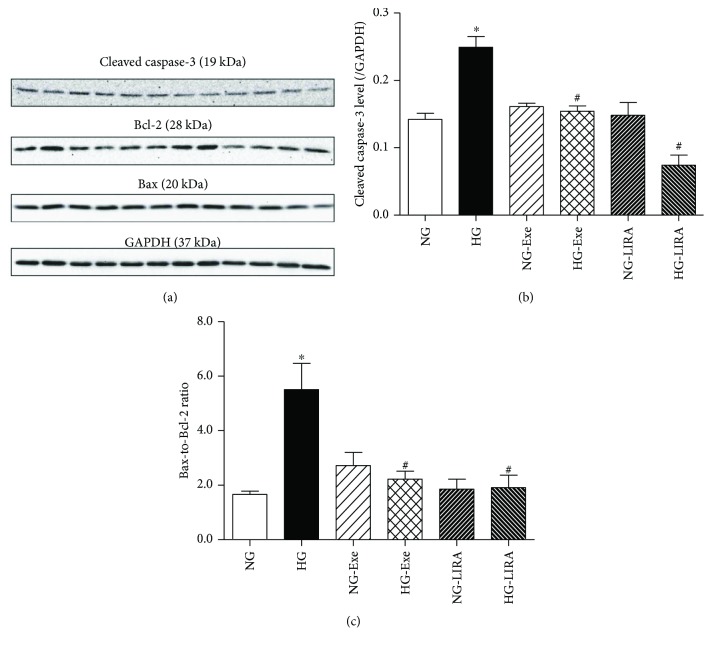
Effect of exendin-4 (Exe) and liraglutide (LIRA) on glucose toxicity-induced elevation of apoptotic proteins: (a) representative gel bands of cleaved caspase-3, Bax, Bcl-2, and GAPDH (loading control) using specific antibodies; (b) quantitative analysis of cleaved caspase-3; (c) quantitative analysis of the Bax-to-Bcl-2 ratio. Mean ± SEM, *n* = 4–6 cultures per group, ^∗^*p* < 0.05 versus the NG group and ^#^*p* < 0.05 versus the HG group.

To determine the role of GLP-1 activation on glucose-induced apoptosis, apoptotic-related proteins including cleaved caspase-3, Bax, and Bcl-2 were evaluated. As shown in Figure 3, high glucose incubation significantly upregulated the levels of cleaved caspase-3 and Bax-to-Bcl-2 ratio, the effects of which were abrogated by Exe and LIRA with little effect from either GLP-1 agonist itself.

### 3.4. Exendin-4 and Liraglutide Ameliorated High Glucose-Induced Loss in MMP

**Figure 4:**
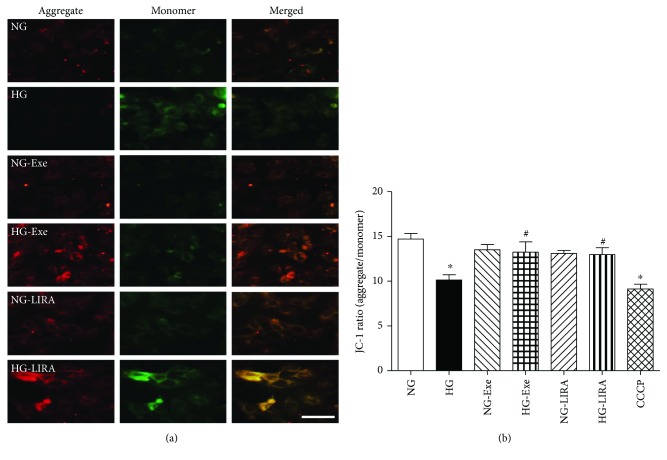
Effect of exendin-4 (Exe) and liraglutide (LIRA) on glucose toxicity-induced loss of mitochondrial membrane potential (MMP) assessed using JC-1: (a) representative fluorescent images of JC-1 staining depicting aggregates (red fluorescence), monomer (green fluorescence), and merged (yellow fluorescence), scale bar = 50 *μ*m; (b) pooled data depicting quantitative analysis of the JC-1 ratio. Mean ± SEM, *n* = 10 images per group, ^∗^*p* < 0.05 versus the NG group and ^#^*p* < 0.05 versus the HG group.

JC-1 staining was performed to determine the stability of MMP in H9c2 cells. Our results revealed that high glucose significantly decreased MMP, as evidenced by the decreased aggregate-to-monomer ratio. Although neither Exe nor LIRA affected MMP levels in the normal glucose group, they effectively nullified the high glucose-induced drop in MMP (Figure 4).

### 3.5. Exendin-4 and Liraglutide Protected against Glucose Toxicity-Induced Loss in Autophagy

**Figure 5:**
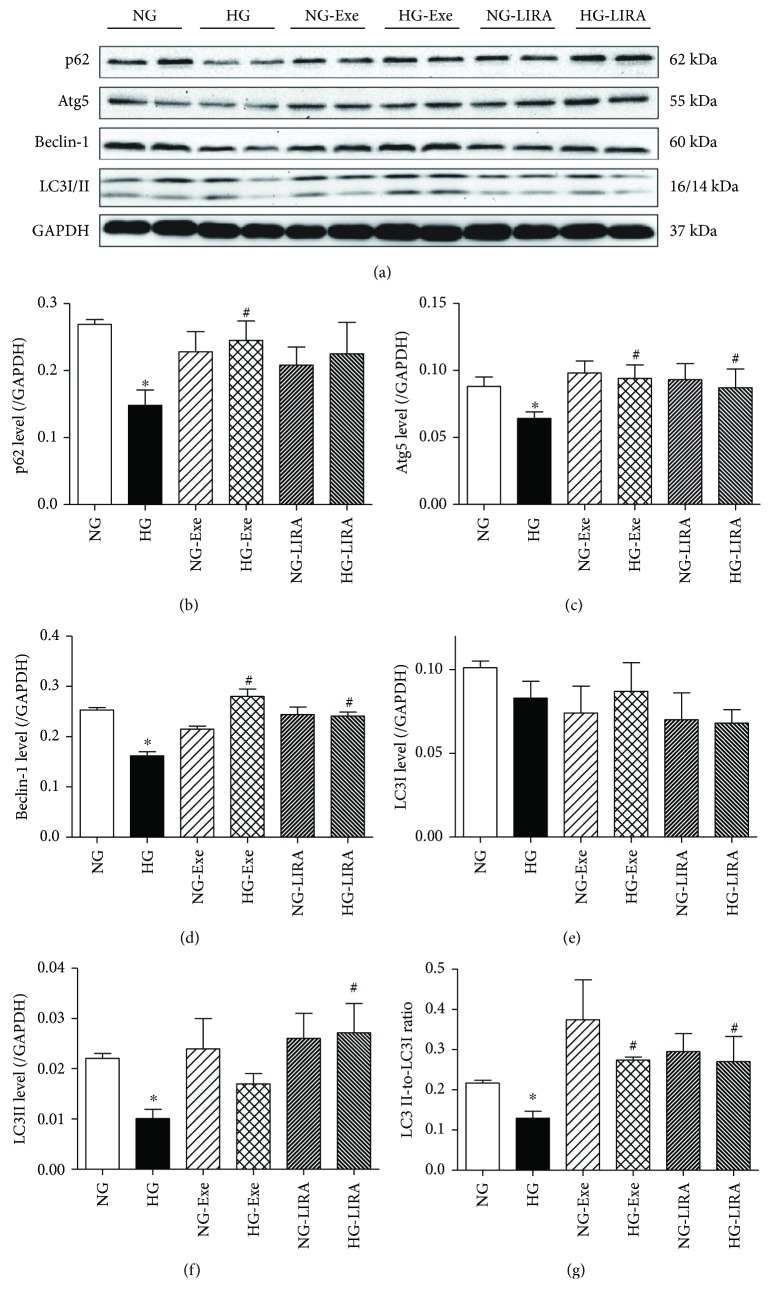
Effect of exendin-4 (Exe) and liraglutide (LIRA) on glucose toxicity-induced loss of autophagy: (a) representative pictures of p62, Beclin-1, Atg5, and LC3I/II using GAPDH as the loading control; (b) p62 levels; (c) Atg5 levels; (d) Beclin-1 levels; (e) LC3I levels; (f) LC3II levels; (g) LC3II-to-LC3I ratio. Mean ± SEM, *n* = 3–6 cultures per group, ^∗^*p* < 0.05 versus the NG group and ^#^*p* < 0.05 versus the HG group.

**Figure 6:**
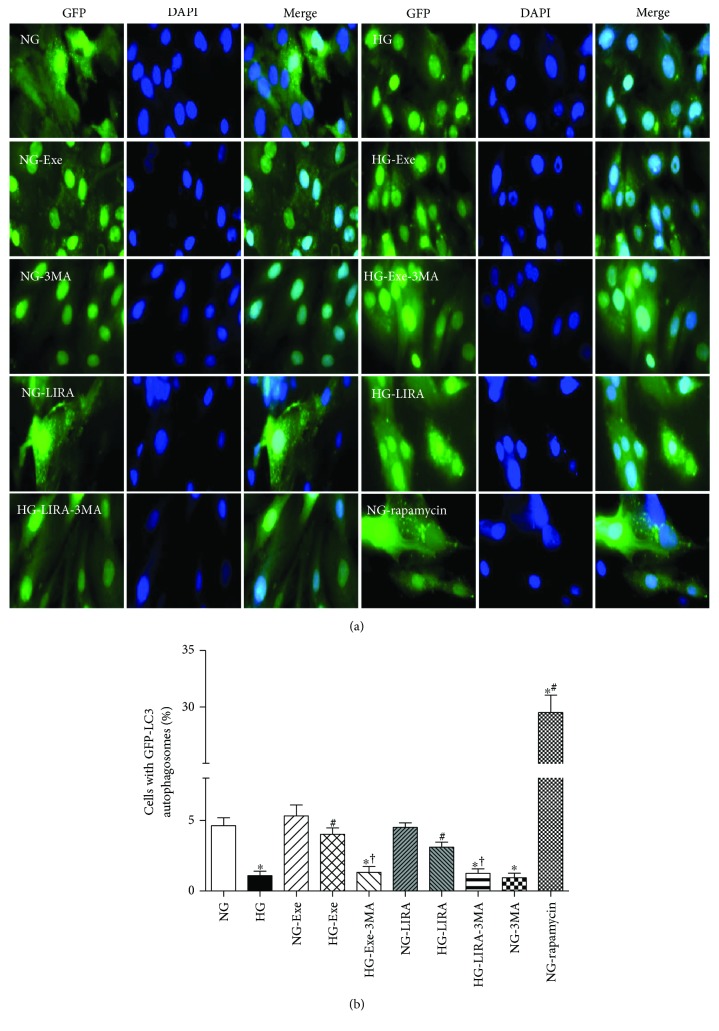
Effect of exendin-4 (Exe) and liraglutide (LIRA) on glucose toxicity-induced loss of GFP-LC3 puncta; (a) representative images of GFP-LC3, DAPI staining and merged images of GFP-LC3, and DAPI staining in H9c2 cells cultured in normal-glucose or high-glucose medium treated with or without Exe, LIRA, the autophagy inhibitor 3MA, or the autophagy inducer rapamycin; (b) quantitative analysis of cells with GFP-LC3 positive puncta. Mean ± SEM, *n* = 10–14 fields of independent cultures per group, ^∗^*p* < 0.05 versus the NG group, ^#^*p* < 0.05 versus the HG group and ^†^*p* < 0.05 versus Exe or LIRA-treated HG group.

Defective autophagy was reported in the setting of diabetes or hyperglycemia and may contribute to the pathogenesis of diabetic cardiomyopathy [11, 30, 32]. To discern the role of autophagy in the GLP-1 activation-induced beneficial role against glucose toxicity, levels of autophagy-related proteins were examined using Western blot analysis. As shown in Figure 5, high glucose challenge (for 48 hours) significantly decreased the levels of autophagy protein markers including Beclin-1, Atg5, LC3II, LC3II-to-LC3I ratio, and p62, the effects of which were negated by Exe and LIRA. Neither GLP-1 agonist produced any notable effect on autophagy protein markers in a normal glucose environment. Next, fluorescence microscopy was employed to visualize autophagosome formation. LC3-II accumulates due to increased autophagosome formation or impaired autophagosome-lysosome fusion. As shown in Figure 6, H9c2 cells cultured in a high-glucose medium exhibited a decrease in the number of punctate GFP-LC3 structures. Although neither GLP-1 agonist had any effect on GFP-LC3 puncta formation, they effectively rescued against a glucose toxicity-induced decrease in the number of punctate GFP-LC3. Furthermore, class III PtdIns3K inhibitor 3-MA, an autophagy inhibitor, ablated Exe- or LIRA-induced restoration of LC3II accumulation. These data strongly indicated that Exe and LIRA protects against high glucose-induced loss in autophagy.

### 3.6. Exendin-4 and Liraglutide Promoted mTOR/ULK1-Dependent Signaling

**Figure 7:**
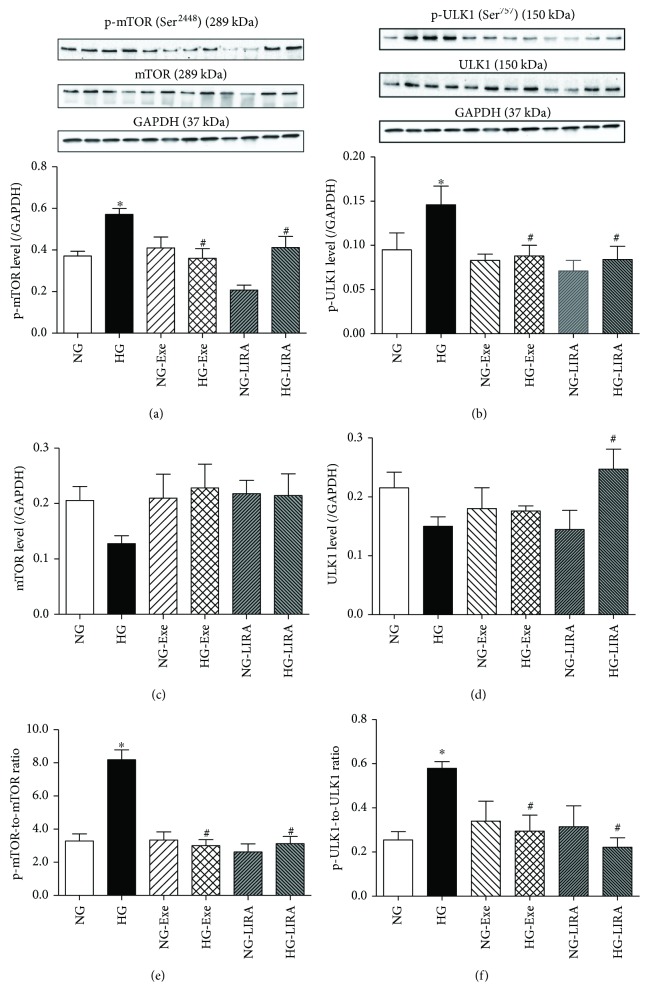
Effect of exendin-4 (Exe) and liraglutide (LIRA) on glucose toxicity-induced changes in the phosphorylation of mTOR and ULK1 in murine cardiomyocytes: (a) p-mTOR levels; (b) p-ULK1 levels; (c) mTOR levels; (d) ULK1 levels; (e) p-mTOR-to-mTOR ratio; (f) p-ULK1-to-ULK1 ratio. Insets: Representative pictures of mTOR, p-mTOR, ULK1, and p-ULK1 using GAPDH as the loading control. Mean ± SEM, *n* = 4–6 per group, ^∗^*p* < 0.05 versus the NG group and ^#^*p* < 0.05 versus the HG group.

To determine whether Exe and LIRA activates autophagy through the classical mTOR/ULK1-dependent pathway, mTOR and ULK1 signaling was examined using Western blot. As demonstrated in Figure 7, high glucose incubation significantly increased phosphorylation of both mTOR and ULK1 in murine cardiomyocytes, the effects of which were negated by Exe and LIRA. Neither GLP-1 agonist exhibited any effect on phosphorylation of mTOR and ULK1. Neither GLP-1 agonist nor glucose challenge overly affected the pan protein expression of mTOR and ULK1. These results demonstrated that mTOR/ULK1 signaling is likely involved in GLP-1 activation-offered restoration of autophagy in the face of high glucose challenge.

### 3.7. Role of mTOR in Exendin-4- and Liraglutide-Induced Cardiomyocyte Mechanical Responses against High Glucose

**Figure 8:**
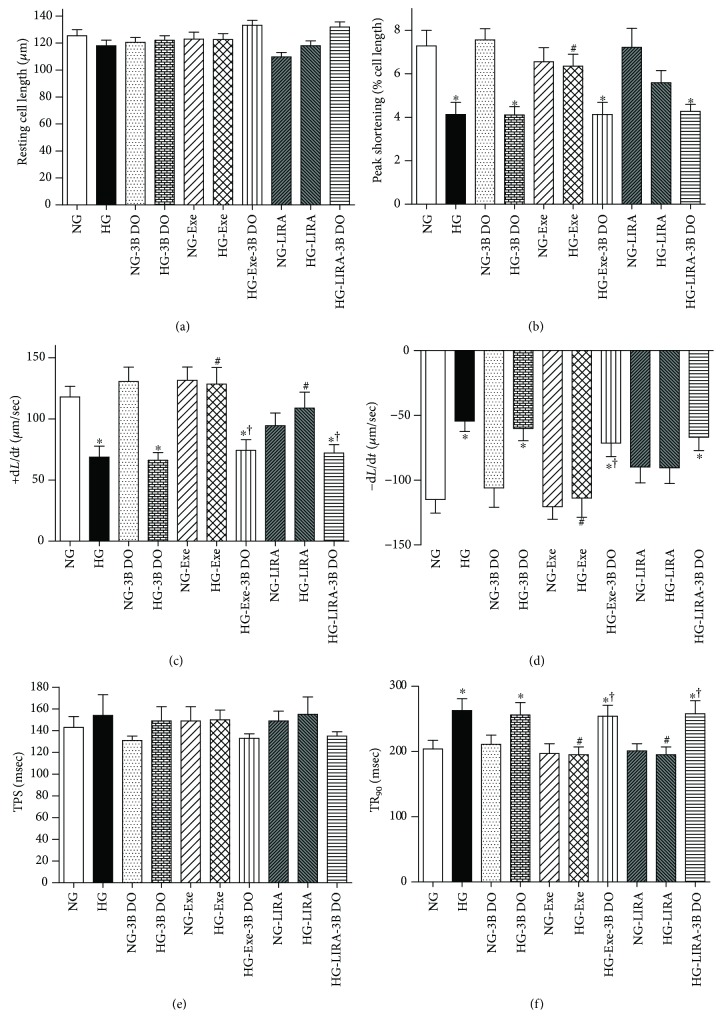
Effect of the mTOR activator 3-benzyl-5-((2-nitrophenoxy) methyl)-dihydrofuran-2(3H)-one (3BDO, 120 *μ*M) on exendin-4- (Exe-) and liraglutide- (LIRA-) induced response against high glucose- (HG-) induced cardiomyocyte mechanical dysfunction in ventricular myocytes isolated from adult mouse hearts: (a) resting cell length; (b) peak shortening (PS); (c) maximal velocity of shortening (+d*L*/d*t*); (d) maximal velocity of relengthening (−d*L*/d*t*); (e) time-to-PS (TPS); (f) time-to-90% relengthening (TR_90_). Mean ± SEM, *n* = 39 cells per group, ^∗^*p* < 0.05 versus the NG group, ^#^*p* < 0.05 versus the HG group, and ^†^*p* < 0.05 versus the corresponding GLP-1 agonist-treated group.

To evaluate whether mTOR plays a permissive role in GLP-1 agonists Exe- and LIRA-induced beneficial response against high glucose challenge, adult murine cardiomyocytes were incubated with normal- (5.5 mM) or high-glucose (33 mM) medium in the absence or presence of GLP-1 agonists and the mTOR activator 3BDO (120 *μ*M) for 4 hours prior to the assessment of cardiomyocyte function. As shown in the Figure 8, the mTOR activator negated Exe- and LIRA-induced protection against high glucose-induced cardiomyocyte mechanical dysfunction (decreased PS, +d*L*/d*t*, and −d*L*/d*t* as well as prolonged TR_90_, with unchanged resting cell length and TPS). mTOR activation itself did not affect any cardiomyocyte mechanical property in cells incubated in normal- or high-glucose medium. These results favored a permissive role for mTOR in GLP-1 agonist-offered beneficial response against glucose toxicity.

## 4. Discussion

The salient findings from our study suggested that the GLP-1 agonists Exe and LIRA protect against short-term high-glucose incubation-induced impairment in cardiac contractile function, ROS/O_2_^−^ production, apoptosis, and mitochondrial injury. Glucose toxicity-induced cardiomyopathy is believed to contribute to ventricular dysfunction and the onset of heart failure in diabetes [33–36]. While treatment of diabetic cardiomyopathy remains challenging, our work suggested that GLP-1, an incretin hormone, may serve as an alternative avenue for cardiovascular complications in diabetes. Our data revealed a likely role for autophagy in GLP-1 activation-offered beneficial effects.

Data from our study suggested that short-term high-glucose challenge compromised cardiac function, promoted accumulation of ROS and O_2_^−^, and triggered apoptosis and loss of MMP, in a manner somewhat similar to our earlier reports [35, 36]. Oxidative stress and apoptosis are considered the main contributing factors in the pathogenesis of diabetic heart anomalies [37]. Our observation of DHE, DCF, and MitoSOX Red staining supported the earlier notion of oxidative stress upon high glucose challenge. Mitochondria usually yield energy through oxidative phosphorylation by way of the electron transport chain although incomplete reduction of O_2_ could result in O_2_^−^ production. Our MitoSOX measurement indicated much higher MitoSOX fluorescence in high glucose-challenged H9c2 cells, the effect of which was attenuated by GLP-1 activation. Our further examination noted overtly decreased levels of Nrf2, an oxidative stress-activated transcription factor to maintain MMP and ATP production [38], upon high glucose challenge. It is likely that lack of Nrf2 reduces adaptation and intrinsic resistance to defend against glucose toxicity-induced oxidative stress and apoptosis (as shown by elevation of caspase-3 and the Bax-to-Bcl-2 ratio). Bcl-2 is an antiapoptotic protein to suppress mitochondrial apoptosis via antagonism of Bax oligomerization and later cytochrome c release [39]. On the other hand, high glucose incubation promoted levels of caspase-3, a terminal proapoptotic effector. These data collectively suggested that Exe and LIRA offer their cardioprotective effects possibly through inhibition of oxidative stress and mitochondria-dependent apoptosis. Involvement of mitochondrial injury in GLP-1 agonist-offered protection against glucose toxicity received further supports by MMP and is in line with the notion of disturbed preservation of MMP by mitochondrial respiratory chain in diabetes [40, 41]. It is plausible to speculate that sustained MMP in the face of Exe and LIRA treatment contributes to the inhibition of ROS generation.

Reduced autophagy leads to the buildup of damaged organelles including mitochondria, which subsequently releases proapoptotic factors and ROS, prompting cardiac dysfunction and the development of diabetic cardiomyopathy [42]. In our study, we noted suppressed autophagy in glucose-challenged cardiomyocytes, the effect of which was restored by GLP-1 agonists Exe and LIRA. This is supported by several experimental data. (1) Autophagosome formation (GFP-LC3 puncta) was drastically dampened by high glucose, the effect of which was reversed by GLP-1 agonists. Interestingly, the GLP-1 activation-elicited autophagosome formation against high glucose was cancelled off by the autophagy inhibitor 3-MA. (2) Western blot data revealed decreased Atg5, Beclin-1, LC3II levels, and LC3II-to-LC3I ratio in the high glucose group, the effect of which was reversed by Exe and LIRA. Endogenous Atg5 and Atg12 are manifested as the Atg12-Atg5 conjugate, vital for autophagy [43]. On the other hand, Beclin-1, a core complex of the class III PI3K, helps to recruit autophagy-related proteins onto the isolation membrane in the autophagy process [44]. LC3, commonly referred to as MAP1LC3, plays a pivotal role in autophagosome formation via conversion of LC3I localized in the cytosol to the autophagosome-bound LC3II. Although the increased p62 levels seem to be somewhat paradoxical to reduced autophagy with high glucose challenge, it is possible that suppressed autophagy or autophagosome formation may end up delivering fewer autophagosomes to be degraded in lysosomes, therefore yielding few autophagolysosomes. With the induction of autophagy by GLP-1 activation, p62 levels were restored as observed in our study.

Our study showed that the inactivation of the mTOR/ULK1-dependent pathway may serve as a key mechanism for the cardiac protective role of Exe and LIRA. This was further supported by the fact that the mTOR activator 3BDO effectively nullified GLP-1 agonist-offered beneficial response against glucose toxicity, favoring a permissive role for mTOR in GLP-1 analogue-offered protection against cardiomyopathy. mTOR serves as the most important negative regulator of autophagy through ULK1 [45]. In our hands, high glucose incubation promoted phosphorylation of mTOR at Ser^2448^, in line with an earlier report [45]. ULK1 serves as a downstream signaling molecule for mTOR where mTOR phosphorylates ULK1 at Ser^757^ to suppress ULK1 activation and subsequently autophagy induction [46]. Our data revealed that GLP-1 analogues effectively reversed high glucose-induced overactivation or phosphorylation of mTOR and ULK1, favoring autophagy induction. These findings support a likely role for the mTOR/ULK1 signaling cascade in the regulation of GLP-1 agonist-elicited autophagic responses in the face of glucose toxicity.

In summary, findings from our study provided convincing evidence that Exe and LIRA rescue against high glucose-induced cardiac contractile function, oxidative stress, mitochondrial injury, and apoptosis possibly via regulation of mTOR and autophagy. Exe and LIRA may offer their beneficial effect through inhibited phosphorylation of mTOR and, subsequently, ULK1 (at Ser^757^ residue). These outcomes should shed some light towards a better understanding of the utility of GLP-1 agonists in diabetes- or hyperglycemia-induced cardiac anomalies, which merit further investigation in a more clinically relevant setting.

## Data Availability

The data used to support the findings of this study are available from the corresponding author upon request.
